# Genetic divergence between two phenotypically distinct bottlenose dolphin ecotypes suggests separate evolutionary trajectories

**DOI:** 10.1002/ece3.3335

**Published:** 2017-09-29

**Authors:** Pedro F. Fruet, Eduardo R. Secchi, Juliana C. Di Tullio, Paulo César Simões‐Lopes, Fábio Daura‐Jorge, Ana P. B. Costa, Els Vermeulen, Paulo A. C. Flores, Rodrigo Cezar Genoves, Paula Laporta, Luciano B. Beheregaray, Luciana M. Möller

**Affiliations:** ^1^ Museu Oceanográfico ‘Prof. Eliézer de C. Rios’ Rio Grande RS Brazil; ^2^ Laboratório de Ecologia e Conservação da Megafauna Marinha – ECOMEGA Instituto de Oceanografia Universidade Federal do Rio Grande (FURG) Rio Grande RS Brazil; ^3^ Programa de Pós‐Graduação em Oceanografia Biológica Universidade Federal do Rio Grande (FURG) Rio Grande RS Brazil; ^4^ Molecular Ecology Laboratory Flinders University Adelaide SA Australia; ^5^ Kaosa Rio Grande RS Brazil; ^6^ Laboratório de Mamíferos Aquáticos (LAMAQ) Departamento de Ecologia e Zoologia Universidade Federal de Santa Catarina (UFSC) Florianópolis SC Brazil; ^7^ Department of Biology University of Louisiana at Lafayette Lafayette LA USA; ^8^ Whale Unit Mammal Research Institute University of Pretoria Hatfield Pretoria South Africa; ^9^ Whalefish Lancefield Quay Glasgow UK; ^10^ APA Anhatomirim‐SC ICMBio Jurerê Florianópolis SC Brazil; ^11^ Yaqu Pacha Uruguay – Organización para la Conservación de Mamíferos Punta del Diablo Rocha Uruguay; ^12^ Centro Universitario Regional del Este Universidad de la República Rocha Uruguay; ^13^ Cetacean Ecology, Behaviour and Evolution Laboratory Flinders University Adelaide SA Australia

**Keywords:** biopsy sampling, conservation, evolutionarily significant unit, microsatellites, mtDNA, South America

## Abstract

Due to their worldwide distribution and occupancy of different types of environments, bottlenose dolphins display considerable morphological variation. Despite limited understanding about the taxonomic identity of such forms and connectivity among them at global scale, coastal (or inshore) and offshore (or oceanic) ecotypes have been widely recognized in several ocean regions. In the Southwest Atlantic Ocean (SWA), however, there are scarce records of bottlenose dolphins differing in external morphology according to habitat preferences that resemble the coastal‐offshore pattern observed elsewhere. The main aim of this study was to analyze the genetic variability, and test for population structure between coastal (*n* = 127) and offshore (*n* = 45) bottlenose dolphins sampled in the SWA to assess whether their external morphological distinction is consistent with genetic differentiation. We used a combination of mtDNA control region sequences and microsatellite genotypes to infer population structure and levels of genetic diversity. Our results from both molecular marker types were congruent and revealed strong levels of structuring (microsatellites *F*_ST_ = 0.385, *p* < .001; mtDNA *F*_ST_ =  0.183, *p* < .001; Φ_ST_ = 0.385, *p* < .001) and much lower genetic diversity in the coastal than the offshore ecotype, supporting patterns found in previous studies elsewhere. Despite the opportunity for gene flow in potential “contact zones”, we found minimal current and historical connectivity between ecotypes, suggesting they are following discrete evolutionary trajectories. Based on our molecular findings, which seem to be consistent with morphological differentiations recently described for bottlenose dolphins in our study area, we recommend recognizing the offshore bottlenose dolphin ecotype as an additional Evolutionarily Significant Unit (ESU) in the SWA. Implications of these results for the conservation of bottlenose dolphins in SWA are also discussed.

## 1 INTRODUCTION

The intraspecific variation is critical for conservation biology because it addresses variability that is relevant for species persistence and evolutionary potential (e.g., Allendorf & Luikart, 2007). The identification of distinct population segments, however, can be a challenging task. This is especially true for highly mobile and widely distributed species inhabiting the marine environment that lacks evident physical barriers to gene flow (Hoelzel, 2009; Palumbi, 1994). Some species might adapt to, and evolve in, different habitats or even in sympatry as a result of feeding specializations, forming so‐called ecotypes, with limited or no contemporary gene flow between them (e.g., Foote, Newton, Piertney, Willerslev, & Gilbert, 2009; Foote et al., 2011; Louis, Viricel, et al., 2014; Louis, Fontaine, et al., 2014; Natoli, Peddemors, & Hoelzel, 2004). Ecotypes may possess unique adaptations and distinct evolutionary histories and hence could be considered as separate Evolutionarily Significant Units (ESU) (Ryder, 1986)—a practical concept widely used for prioritizing management actions within species (Moritz, 1994, 1999).

Inferring population structure using molecular markers is a powerful tool for identifying distinct populations for management (Allendorf & Luikart, 2007). Over the past decades, the use of genetic markers has increased substantially in cetacean studies, revealing varying levels of populations structuring over large and small spatial scales for some species (e.g., Natoli et al., 2004; Pérez‐Alvarez et al., 2017; Rosel, Hansen, & Hohn, 2009). The molecular approach, when integrated with phenotypic and ecological data, has proven to provide reliable information for cetacean taxonomic diagnosis and for understanding evolutionary forces shaping genetic divergence (e.g., Caballero et al., 2007; Cunha et al., 2015; Louis, Fontaine, et al., 2014; Wang, Chou, & White, 1999).

The common bottlenose dolphin (*Tursiops truncatus*) is a cosmopolitan cetacean species adapted to a wide range of environments. Such plasticity makes the species to vary geographically in a significant number of biological traits. Despite limited understanding about the taxonomic identity of geographical forms and connectivity among populations, coastal (or inshore) and offshore (or oceanic) ecotypes have been widely recognized in several ocean regions (Hoelzel, Potter, & Best, 1998; Mead & Potter, 1995; Perrin, Thieleking, Walker, Archer, & Robertson, 2011; Tezanos‐Pinto et al., 2009; Van Waerebeek, Reyes, Read, & McKinnon, 1990). In the North Atlantic, for example, coastal and offshore ecotypes are notably distinct in their genetic profiles and several other morphological and biological aspects (e.g., Hersh & Duffield, 1990; Hoelzel et al., 1998; Mead & Potter, 1995). In general, the coastal ecotype is smaller, lighter gray, and forms small fragmented populations, while the offshore ecotype is larger, darker, and forms larger populations of up to thousands of individuals connected over broad geographical scales (see Wells & Scott, 2009). Results of some regional studies investigating the ecotypes differentiation have reported marked differences in genetics, morphology, and feeding habits between the ecotypes in the Northeastern Pacific (e.g., Mead & Potter, 1995; Perrin et al., 2011; Walker, 1981) and Northwestern Atlantic (e.g., Hoelzel et al., 1998). In the Northeastern Atlantic, bottlenose dolphin ecotypes also form two clear genetically distinct groups, despite the lack of evident external morphological differences (Louis, Viricel, et al., 2014; Louis, Fontaine, et al., 2014).

Along the Southwest Atlantic Ocean (SWA), bottlenose dolphins occur in both coastal and offshore zones. In coastal regions, they are commonly seen in shallow waters (<20 m) within 3 km from the coast (e.g., Di Tullio, Fruet, & Secchi, 2015; Laporta et al., 2016). The occurrence of coastal populations is restricted to southeastern and southern Brazil (27°S) down to central Argentina (43°S), despite some records of sporadic movements beyond these limits (e.g., Simões‐Lopes & Fábian, 1999). These coastal populations are small (<100 individuals) and associated with estuaries, river mouths, and lagoons (see Simões‐Lopes, 1991; Lodi et al., 2016; Laporta, et al., 2016; Fruet, Flores, & Laporta, 2016; Fruet et al., 2016; for reviews). Sighting data suggest no movement of coastal bottlenose dolphins to deep waters (i.e., >20 m depth), although movements of individuals between coastal areas frequently occur (Würsig, 1978; Simões‐Lopes & Fábian, 1999; Laporta, Di Tullio, et al., 2016; Laporta, Fruet, & Secchi, 2016). Recent studies have shown remarkably low levels of genetic diversity and strong genetic differences among these coastal populations at both microsatellites and mitochondrial DNA markers (Costa et al., 2015; Fruet et al., 2014). At a larger geographical scale, it was suggested that bottlenose dolphins, from Bahía San Antonio (BSA), Argentina, and southern Brazil–Uruguay (SBU), form two distinct ESUs with negligible contemporary gene flow between them (Fruet et al., 2014). Additional subdivisions were also found for the SBU‐ESU, consisting of multiples management units (Fruet et al., 2014). On the other hand, sightings of bottlenose dolphins in offshore waters in the SWA are reported mainly beyond the continental shelf break (>150 m of depth), and approximately 100 km or further from the coast (e.g., Di Tullio, Gandra, Zerbini, & Secchi, 2016). Despite little information being available for bottlenose dolphins in offshore waters, observational data and photographs taken in these waters suggest clear differences in external morphology and coloration patterns in relation to coastal bottlenose dolphins (P.C. Simões‐Lopes, personal observation; Laboratório de Ecologia e Conservação da Megafauna Marinha, unpublished data; see supporting information of Costa, Rosel, Daura‐Jorge, & Simões‐Lopes, 2016). These patterns are similar to the coastal‐offshore pattern observed elsewhere (e.g., Hersh & Duffield, 1990; Van Waerebeek et al., 1990).

In this study, we analyze genetic variability and test for population structure between coastal and offshore bottlenose dolphins sampled in the SWA to assess whether external morphological distinction is associated to genetic differences. These data, in conjunct with previous information, allowed reassessing the population structure of common bottlenose dolphins in a broader geographical context in the SWA, with results leading to the proposal of a new ESU for the species in this region.

## 2 METHODS

### 2.1 Sample collection and stratification

**Figure 1:**
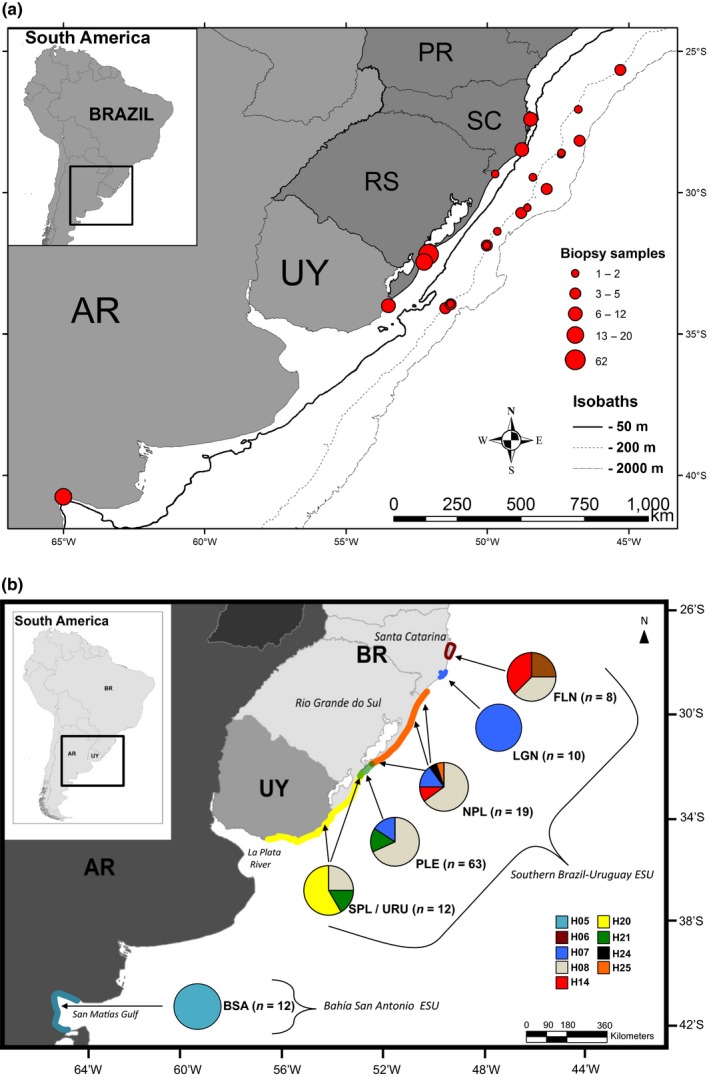
Study area in the Southwest Atlantic Ocean. (a) Sampling sites of common bottlenose dolphins (*Tursiops truncatus*) in coastal and offshore waters, where AR, Argentina; UY, Uruguay; RS, state of Rio Grande do Sul; SC, state of Santa Catarina; PR, state of Paraná; (b) Figure modified from Fruet et al. (2014) showing the proposed Evolutionarily Significant Units (ESUs) and Management Units (MUs) (color counter lines) for the coastal ecotype, and the respective frequencies of mitochondrial control region haplotypes (pie charts). Arrows indicate the main sampling locations. FLN, Florianópolis; LGN, Laguna; NPL, north Patos Lagoon; PLE, Patos Lagoon estuary; SLP/URU, south Patos Lagoon/Uruguay; BSA, Bahía San Antonio

The study area covers approximately 2,100 and 1,000 km of linear distance in coastal and oceanic waters of the SWA, respectively. It extends from the state of Paraná (PR), in southern Brazil, to Bahía San Antonio (BSA), in the Patagonian Argentina (25°18–54°40′S) (Figure 1). Along this region, biopsies were taken from common bottlenose dolphins using modified darts specifically designed for small cetaceans (F. Larsen, Ceta‐Dart) fired from a 120‐lb draw weight crossbows.

**Figure 2:**
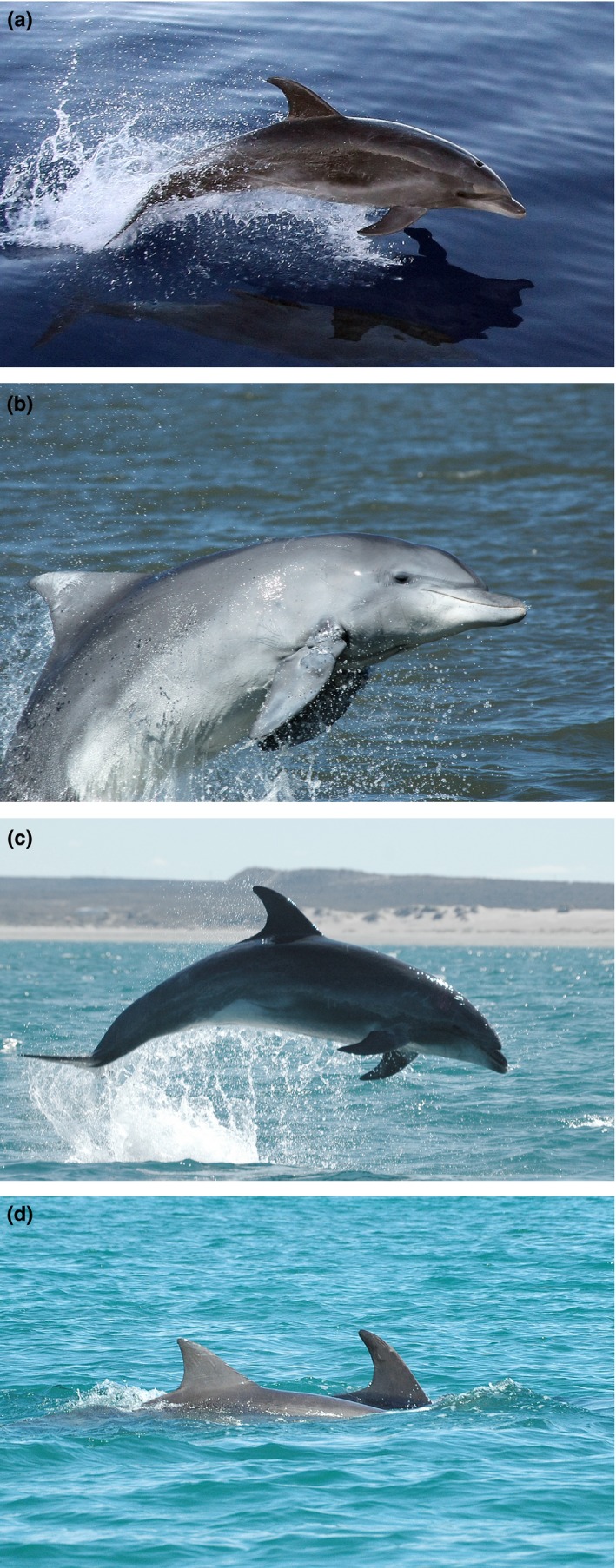
Differences in external morphology and coloration between offshore and coastal bottlenose dolphin ecotypes (*Tursiops truncatus*) in the Southwest Atlantic Ocean. (a) Offshore bottlenose dolphin ecotype photographed during biopsy sampling activities in the outer continental shelf of southern Brazil (Photo credit: Luciano Dalla Rosa). (b) Typical coastal resident bottlenose dolphin in Patos Lagoon estuary, southern Brazil. Note the light gray coloration, triangular dorsal fin, and relatively longer beak. (c) Bottlenose dolphin photographed in Bahía San Antonio, Argentina, resembling the putative offshore ecotype. Note the short beak and falcate dorsal fin. (d) Differences in dorsal fin shape and coloration of sympatric putative offshore and coastal ecotypes of bottlenose dolphins in Bahía San Antonio, Argentina

In the offshore waters, 45 biopsies from 15 different groups were taken during eight ship‐based surveys carried out during spring and autumn between 2009 and 2012 on the outer continental shelf (~150 m isobath) to the slope (up to the 1,500 m isobath) off southeast and southern Brazil (~23°S to ~34°S) (Di Tullio et al., 2016). All samples were collected in water depths greater than 146 m (mean = 412 m) and minimal distance of 103 km from the coast (mean = 143 km). All bottlenose dolphin biopsies collected during these ship‐based surveys were morphologically distinct from coastal dolphins (darker in coloration, falcate dorsal fin, and with apparent shorter beak, Figure 2) and thus were considered to belong to a putative offshore ecotype. There was no sampling effort in the offshore waters off Uruguay and Argentina.

With the exception of three additional samples collected from dolphins regularly sighted in BSA that are morphologically distinct from their conspecifics, and resemble those of the putative offshore ecotype (Bastida, Rodríguez, Secchi, & da Silva, 2007; Vermeulen & Cammareri, 2009a – see Figure 2c), samples from coastal bottlenose dolphins (*n* = 124) are the same used in a recent study that investigated the fine‐scale genetic structuring of these dolphins in the SWA (Fruet et al., 2014). In brief, 120 biopsies were collected between 2004 and 2012 during small boat‐based surveys conducted in coastal, shallow waters (<2 km from shore, <10 m deep) of southern Brazil, Uruguay, and Argentina. Biopsies were taken from individuals of six well‐studied dolphin communities (four in Brazil, one in Uruguay, and one in Argentina). These coastal bottlenose dolphins display a smaller and triangular dorsal fin and a relatively longer beak and a light gray coloration than offshore bottlenose dolphins (Figure 2b,d). Four samples of freshly stranded carcasses of photo‐identified dolphins completed the final dataset (see Fruet et al., 2014 for more details). Fruet et al. (2014) proposed the existence of two distinct ESUs of coastal bottlenose dolphins in the SWA: one comprising a metapopulation of five communities along the Southern Brazil–Uruguay (SBU‐ESU), and another including the Bahía San Antonio dolphin community, Argentina (BSA‐ESU) (see Figure 1 for details). Thus, the final dataset of the coastal ecotype consisted of 15 samples from the BSA [12 previously analyzed by Fruet et al. (2014) and three additional samples in this study] and 112 from the SBU‐ESUs.

All samples used in this study (offshore and coastal) were preserved in 20% dimethyl sulphoxide (DMSO) saturated with sodium chloride (Amos & Hoelzel, 1991) or 98% ethanol, and followed identical laboratory procedures.

### 2.2 DNA extraction and molecular sexing

Samples were processed at the Molecular Ecology and Evolution Lab, Flinders University, South Australia. DNA was extracted following a salting‐out protocol (Sunnucks & Hales, 1996), and molecular sexing was determined by the amplification of fragments of the SRY and ZFX genes through the polymerase chain reaction (PCR) using the protocol developed by Gilson, Syvanen, Levine, and Banks (1998).

### 2.3 mtDNA sequencing and haplotypes definition

We successfully aligned 457 bp of the mtDNA control region (the same fragment used by Fruet et al. (2014) to investigate the population structure of coastal bottlenose dolphins in SWA) for 45 samples collected in offshore waters, plus three samples collected in BSA, Argentina. Sequencing was carried out on an ABI 3730 (Applied Biosystems) automated DNA sequencer according to manufacturer's instructions. Details for mtDNA PCR and sequencing procedures are found in Möller and Beheregaray (2001). To account for potential errors, a total of 10% of samples were resequenced. Sequences were edited using SEQUENCHER 3.0 (Gene Codes Corporation, Ann Arbor, MI, USA). Alignment was run together with the 124 sequences of coastal dolphins available for SBU and BSA‐ESUs (see Fruet et al., 2014) using the ClustalW algorithm in MEGA 5.05 (Tamura et al., 2011) and rechecked by eye. Haplotypes were defined using DNASP 5.0 (Librado & Rozas, 2009) and stored in GenBank under accession number MF405801–MF405833.

### 2.4 Microsatellite genotyping

All 48 samples were subsequently used for microsatellite amplification. Samples were genotyped at 16 microsatellite loci (same used by Fruet et al. (2014) for coastal bottlenose dolphins) with GenScan 500 LIZ 3130 internal size standard. Procedures for microsatellite PCR and genotyping are found in Möller and Beheregaray (2004) and Amaral et al. (2012). For microsatellites, bins for each locus were determined and genotypes scored in GENE MAPPER 4.0 (Applied Biosystems). Rare alleles (i.e., frequency <5%) or alleles that fell in between two bins were regenotyped. Micro‐Checker 2.2.3 (Van Oosterhout, Hutchinson, Wills, & Shipley, 2004) was used to check for potential scoring errors, the presence of null alleles, stuttering, and large allelic dropout. Genotyping error rates were estimated by regenotyping eight randomly selected samples, representing ~17% of the total sample size (*n* = 48). We used GenAlEx 6.5 (Peakall & Smouse, 2012) to find potential matches between genotypes. Samples matching at all genotypes or those mismatching at only a few alleles (1–2) were double‐checked for potential scoring errors. Samples sharing identical genotypes, mtDNA haplotype, and sex were considered as resampled individuals, and we retained only one of each of those identified pairs. Genotyping data were deposited in the Dryad digital repository (provisional DOI: https://doi.org/10.5061/dryad.t130r).

### 2.5 Clustering analysis

We used STRUCTURE 2.3 (Pritchard, Stephens, & Donnelly, 2000) to run a Bayesian model‐based clustering to infer population structure based on microsatellite genotyping for a final dataset of 172 samples (48 from this study plus 124 from Fruet et al., 2014). This model calculates the log‐likelihood value of the data to determine the most likely number of clusters (*K*). Individual membership coefficient (q) to each cluster is also estimated providing valuable information on the similarity between individuals based on shared ancestry. We assumed correlated allele frequencies (Falush, Stephens, & Pritchard, 2003) and an admixture model with no a priori information (Hubisz, Falush, Stephens, & Pritchard, 2009). Simulations were performed using 200,000 burn‐in and 10^6^ repetitions of the Markov Chain Monte Carlo (MCMC), assuming values of *K* varying between 1 and 4 (two coastal ESUs, one putative offshore population, plus one). As recommended by Gilbert et al. (2012), we performed 20 independent runs to limit the influence of stochasticity and to increase the precision of the parameter estimates. The method of Evanno, Regnaut, and Goudet (2005), which determines the second‐order rate of change of the likelihood function on *K* (∆*K*), was used to determine the most likely value of *K* over multiple runs, as implemented in STRUCTURE HARVESTER (Earl & vonHoldt, 2012 **)**. The Evanno method was used because it reveals the highest hierarchical level of structure, which seems appropriate to test for genetic differentiation between ecotypes of bottlenose dolphins.

### 2.6 Genetic diversity and population structure within and between STRUCTURE clusters

Genetic diversity was assessed within and between clusters inferred by STRUCTURE. For mtDNA, genetic diversity was assessed by estimating haplotype (*h*) and nucleotide diversities (π) (Nei, 1987) using ARLEQUIN 3.5.1.2 (Excoffier & Lischer, 2010). For microsatellites, genetic diversity was expressed as the number of alleles (*N*_A_), expected (*H*_E_) and observed (*H*_O_) heterozygosity, and inbreeding coefficient (*F*_IS_), and was calculated using GenoDive 2.0 (Meirmans & Van Tienderen, 2004). Departures from Hardy–Weinberg equilibrium and linkage disequilibrium were tested using the Fisher's exact test and a Markov chain method with 1,000 iterations in GENEPOP on the web (Raymond & Rousset, 1995). Corrected allelic richness (*A*_R_) per population was estimated in FSTAT 2.9.3.2 (Goudet, 2002). All statistical tests followed sequential Bonferroni correction to address the chance of increased Type I error associated with multiple tests (Rice, 1989). Conventional pairwise *F*‐statistics tests (Weir & Cockerham, 1984; *F*_ST_ and Φ_ST_ for mtDNA, and only *F*_ST_ for microsatellites) were performed to assess population structure between inferred clusters using ARLEQUIN 3.5.1.2 (Excoffier & Lischer, 2010). For Φ_ST,_ we used the Tamura and Nei (1993) model with a gamma correction of 0.5. Significance was tested based on 10,000 permutations. Additionally, we used GenAlEx 6.5 (Peakall & Smouse, 2012) to run a principal coordinate analysis (PCoA) based on the allele frequencies of microsatellites to visually interpret genetic similarities between individuals without the constraint of forcing them into a set of clustering subdivisions. A median‐joining network implemented in the program PopArt (Leigh & Bryant, 2015) was constructed for the visualization of the genealogical relationships among the mtDNA haplotypes.

## 3 RESULTS

### 3.1 Summary statistics

Microsatellite genotypes and mtDNA sequences were identical in replicated samples (i.e., null error rate), and no samples were identified as duplicates for the 48 new samples analyzed in this study. Thus, the final new dataset consisted of 25 males and 20 females for offshore samples and two males and one female for the three dolphins sampled along the coast of BSA (Table 1). Examination of the microsatellite genotypic data across all loci, after Bonferroni correction, for the offshore samples only, revealed significant deviations from Hardy–Weinberg expectations (HWE). The analysis in Micro‐Checker indicated five loci (Tur91, TexVet, EV37, MK8, and KW2) to have potential null alleles in the offshore samples, which were likely results of the HWE tests (Table S1). Therefore, we excluded these five loci from the remaining analyses. We found a nonsignificant inbreeding coefficient as estimated on these 11 loci for the offshore ecotype (*F*_IS_ = 0.05, *p* = .02). No linkage disequilibrium was found between any locus pair.

**Table 1:** Summary of genetic diversity for coastal and offshore common bottlenose dolphin ecotypes (*Tursiops truncatus*) in the Southwest Atlantic Ocean based on a 457 bp fragment of the mtDNA control region and 11 microsatellite loci. Number between brackets indicates total sample size used for estimate genetic diversity (separated by sex). The three individuals sampled in coastal waters of BSA, which were morphologically and genetically identified as offshore ecotype, were excluded from genetic diversity analyses. Measures of genetic diversity for the coastal ecotype are the same reported in Fruet et al. (2014), with the exception of microsatellites because here only 11 loci were included

	mtDNA					Microsatellites					
	Hap.	*s*	Indels	*h*	π	*P*_A_	*N*_A_	**A**_R_	*H*_E_	*H*_O_	*F*_IS_
Offshore (20F:25M)	22	38	2	0.940 (0.016)	0.019 (0.010)	4.8	8.2	7.1	0.65	0.65	0.05
Coastal (61F:63M)	11	18	0	0.702 (0.034)	0.009 (0.005)	1.6	3.3	3.1	0.21	0.26	0.20^a^

*Hap* number of haplotypes*; S* polymorphic sites*; h* haplotype diversity; π nucleotide diversity*; P*_A_ number of private alleles; *N*_A_ mean number of alleles per locus; *A*_R_ mean allelic richness; *H*_E_ mean expected heterozygosity; *H*_O_ mean observed heterozygosity; *F*_IS_ inbreeding coefficient.

a Significant multilocus *p* value (*p* < .001).

### 3.2 Inferred clusters

**Figure 3:**
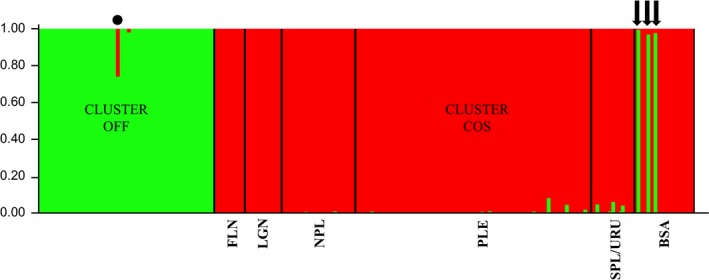
STRUCTURE bar plot of the likelihood (*Y*‐axis) of each individual's (*X*‐axis) assignment to a particular genetic cluster with best estimate for *K* = 2 populations when applying the Evanno method (Evanno et al., 2005). Vertical black lines in Cluster “COS” separate sampled coastal bottlenose dolphin communities. Cluster “OFF” (green vertical lines) contains all common bottlenose dolphins (*Tursiops truncatus*) collected in offshore waters of the SWA, while cluster “COS” (red vertical lines) holds coastal dolphins from SBU and BSA‐ESUs (see Fruet et al., 2014 for details). Black circle in cluster “OFF” indicates an admixed individual. Each arrow in cluster “COS” indicates the three biopsied dolphins in Bahía San Antonio, Argentina, which morphologically resemble offshore bottlenose dolphins and are likely migrants to the coastal population. Black lines separate sampled coastal bottlenose dolphin communities as presented in Fruet et al. (2014): (i) Florianópolis, (ii) Laguna, (iii) north of Patos Lagoon, (iv) Patos Lagoon estuary, (v) south of Patos Lagoon/Uruguay, and (vi) Bahía San Antonio

Results of the STRUCTURE Bayesian clustering analyses based on 11 microsatellite loci showed a strong pattern of population structure with the best estimate for *K* = 2 when applying the Evanno method for the genetic profile of 172 dolphins, including coastal and offshore ecotypes (Fig. S1). Results were highly consistent across runs, and assignment probabilities for all individuals to their clusters were above 0.98, with the exception of six coastal individuals with weak signs of admixture (Figure 3). One individual assigned to cluster “OFF” (sampled in offshore waters) showed strong signal of admixture with the “COS” cluster. Previous analyses had shown strong genetic differentiation between SBU and BSA bottlenose dolphins when running STRUCTURE separately for the same subset of samples from coastal dolphins. Therefore, the following results of population structure and genetic diversity are presented considering offshore and coastal dolphins as different populations, with further proposed subdivision for the coastal ecotype (see methods).

### 3.3 Population structure

#### 3.3.1 Microsatellites

**Figure 4:**
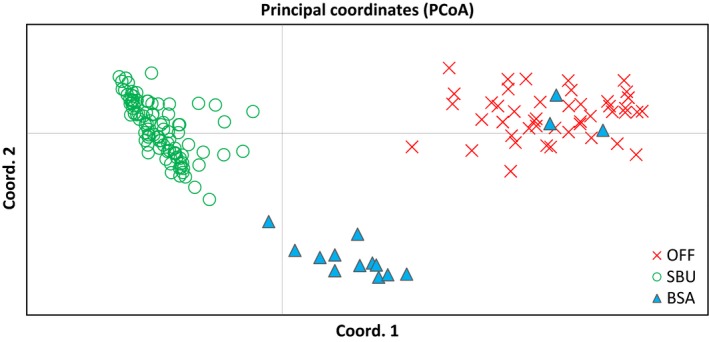
Scatter plot of PCoA scores of genetic similarity among common bottlenose dolphins (*Tursiops truncatus*) from the Southwest Atlantic Ocean based on the allelic frequencies of 11 microsatellite loci. OFF (green x), samples from dolphins collected in offshore waters; SBU (open red circle) and BSA (blue triangles) represent dolphins from coastal southern Brazil–Uruguay and Bahía San Antonio Evolutionarily Significant Units, respectively, which were previously proposed by Fruet et al. (2014)

The results of principal coordinate analysis (PCoA) based on the analysis of 11 microsatellite loci confirmed the patterns of genetic structure revealed by STRUCTURE, with all offshore dolphins grouped toward one side of the ordination plot and the first and second axis explaining 54.4% and 17.5% of variation, respectively (Figure 4). PCoA analysis also assigned the three new samples of individuals collected in BSA‐ESU to the offshore ecotype. The same individual identified in STRUCTURE with a strong sign of admixture was placed between clusters. Additional subdivision was also marked among coastal samples, with BSA and SBU grouping closer to each other than to offshore samples, but with a clear separation between them.

Geographical structuring between ecotypes was also evident and highly significant in the pairwise microsatellite *F*_ST_ population comparisons (*F*_ST_ = 0.385, *p* < .001). High genetic differentiation was also observed when comparing each of the coastal ESU with the offshore ecotype, and *F*_ST_ was higher for offshore‐SBU than for offshore‐BSA comparisons (Table 2).

**Table 2:** Pairwise comparisons of genetic differentiation between coastal and offshore common bottlenose dolphin ecotypes (*Tursiops truncatus*) in the Southwest Atlantic Ocean based on 11 microsatellite loci. Pairwise comparisons between the offshore population and the two proposed Evolutionarily Significant Units (ESUs) for the coastal ecotype (Fruet et al., 2014) are also shown

	Offshore	Coastal	SBU‐ESU	BSA‐ESU
Offshore	0.000			
Coastal	0.385^a^	0.000		
SBU‐ESU	0.415^a^	–	0.000	
BSA‐ESU	0.300^a^	–	0.504^a^	0.000

SBU‐ESU, Southern Brazil–Uruguay; BSA‐ESU, Bahía San Antonio.

Differentiation is expressed as *F*_ST_.

a *p *<* *.001.

#### 3.3.2 mtDNA

Both Φ_ST_ and *F*_ST_ pairwise comparisons for mtDNA data confirmed the pattern of population structure indicated by the nuclear DNA analysis, with coastal bottlenose dolphins highly and significantly differentiated from those inhabiting offshore waters (*F*_ST_ =  0.1829, *p* < .01; Φ_ST_ = 0.385, *p* < .01). Results were similar for both Φ_ST_ and *F*_ST_, but in general, Φ_ST_ had greater values differentiating populations. The highest levels of differentiation were found between SBU and offshore and between SBU and BSA when considering Φ_ST_ and *F*_ST_, respectively (Table 3).

**Table 3:** Pairwise comparisons of genetic differentiation between coastal and offshore common bottlenose dolphin ecotypes (*Tursiops truncatus*) in the Southwest Atlantic Ocean based on 457 bp of the mtDNA control region. Pairwise comparisons between the offshore population and the two proposed Evolutionarily Significant Units (ESUs) for the coastal ecotype (Fruet et al., 2014) are also shown

	Offshore	Coastal	SBU‐ESU	BSA‐ESU
Offshore	0.000	0.385^a^	0.403^a^	0.272^a^
Coastal	0.183^a^	0.000	–	–
SBU‐ESU	0.223^a^	–	0.000	0.262^a^
BSA‐ESU	0.295^a^	–	0.444^a^	0.000

SBU‐ESU, Southern Brazil–Uruguay; BSA‐ESU, Bahia San Antonio.

Differentiation is expressed as Φ_ST_ (above diagonal) and *F*_ST_ (below diagonal).

a *p *<* *.001.

### 3.4 Genetic diversity

#### 3.4.1 Microsatellites

Overall genetic diversity at both nuclear and mtDNA differ between ecotypes (Table 1). For microsatellites, the mean number of alleles per locus was 3.3 in coastal and 8.2 in the offshore dolphins. Allelic richness, a measure that takes sample size into account, was twice as higher for offshore than for coastal bottlenose dolphins. Mean observed heterozygosity showed a similar pattern of variation and was lower than the expected for both ecotypes. Offshore dolphins displayed a high average number of private alleles per locus, but few in high frequency (i.e., >10%—data not shown).

#### 3.4.2 mtDNA

**Figure 5:**
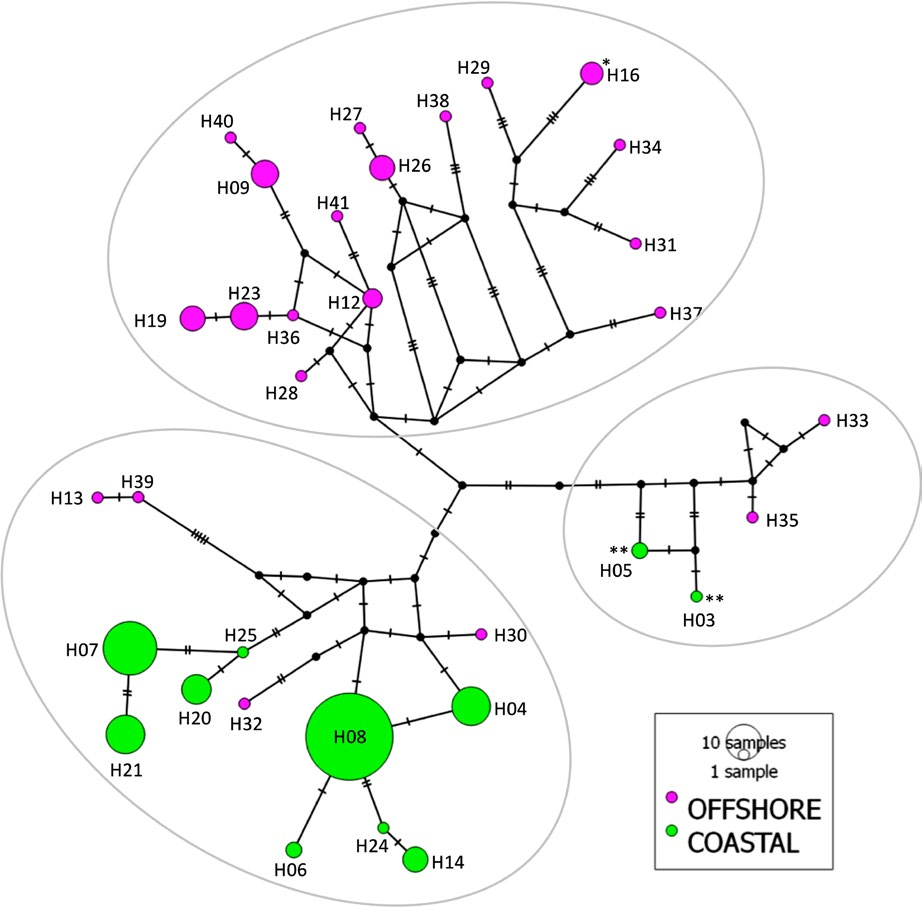
Median‐joining network of haplotypes identified from the analyses of a fragment of the mtDNA control region (457 bp) in coastal and offshore common bottlenose dolphin ecotypes (*Tursiops truncatus*) from the Southwest Atlantic Ocean. Light gray ellipses separate the three main groups of haplotypes. Different colors denote dolphins collected in offshore and coastal waters. Black dots represent extinct or unsampled haplotypes, while dashes represent the number of mutations between haplotypes. *Haplotype of the individual identified with strong sign of admixture in nuclear DNA (see results for STRUCTURE and PCoA analyses for microsatellites). *^*^Haplotypes of individuals (*n* = 3) resembling the offshore ecotype but sampled in coastal waters of Bahía San Antonio, Argentina

Mitochondrial control region sequences of the 457 bp aligned for the 172 samples revealed 33 haplotypes defined by 44 polymorphic sites and two indels (Table 1). Indels were exclusively found in offshore dolphins. There was no haplotype sharing between ecotypes. Haplotype frequencies were highly variable, with offshore dolphins revealing several single haplotypes whereas coastal dolphins displayed few haplotypes at high frequencies (Figure 5). Very low nucleotide and moderate haplotype diversity were found for the coastal ecotype (Table 1). The median‐joining network showed three main haplogroups enclosing the following: (A) only dolphins collected in offshore waters (*n* = 41); (B) all coastal samples plus four offshore dolphins (*n* = 128), with two of the later grouping closely to the most common coastal haplotype; and (C) two offshore dolphins plus the three individuals resembling the offshore ecotype sampled in coastal waters of Argentina (Figure 5). Offshore dolphins displayed highly divergent haplotypes, with a minimum of seven mutational steps separating offshore haplogroups. Twenty‐four mutational steps separated the two most distant haplotypes identified for dolphins collected in offshore waters.

## 4 DISCUSSION

To our knowledge, this is the first study that explicitly tested for genetic differentiation between bottlenose dolphins sampled in distinct habitats (coastal vs. offshore) in the SWA and estimated genetic diversity for offshore dolphins from this region. We found strong levels of structuring and contrasting levels of genetic diversity between the two bottlenose dolphin ecotypes. Results were concordant for mitochondrial and microsatellite DNA markers, supporting patterns found in previous broad‐scale studies that used similar markers (as described below). Results from the Bayesian clustering method implemented in STRUCTURE (with no a priori information) and the PCoA analysis were highly congruent, suggesting that the strong genetic differentiation is not linked to analytical artifacts potentially produced by significant inbreeding coefficients. For the coastal ecotype, a significant deviation of HWE may be due to a combination of further substructuring (coastal ESU's and multiple management units identified) as well as inbreeding in one of the populations (see Fruet et al., 2014).

### 4.1 Genetic diversity

The overall genetic diversity was higher at both marker types in the offshore dolphins of the SWA. Particularly, mtDNA haplotype and nucleotide diversities (*h *=* *0.940; π = 0.019; *n* = 45) were higher than that reported for the offshore ecotype in a worldwide perspective (*h *=* *0.880; π = 0.028; Tezanos‐Pinto et al., 2009) and slightly higher than reported for pelagic Northeast Atlantic (*h *=* *0.929; π=0.014; *n* = 101) and for Mediterranean (*h *=* *0.902; π = 0.013; *n* = 51) bottlenose dolphins (Louis, Viricel, et al., 2014). Similarly, high genetic variation was observed across the 11 microsatellite loci, mirroring the overall pattern reported for offshore ecotypes of bottlenose dolphins worldwide (e.g., Hoelzel et al., 1998; Louis, Viricel, et al., 2014). Within our study region, we found that the offshore ecotype had higher values for all measures of genetic diversity compared to the coastal ecotype, with levels of genetic diversity being nearly three times higher for the offshore dolphins. Such differences in genetic diversity are likely reflecting their contrasting demography, as neutral markers such as mtDNA and microsatellites can have a strong relationship with population size. High levels of genetic diversity typically represent a large panmictic population of thousands of individuals, as it was reported to the offshore bottlenose dolphins in the Northeast Atlantic, which displayed high gene flow and no population structure (Louis, Viricel, et al., 2014; Quérouil et al., 2007). This seems to be in agreement with reports of sighting data from systematic ship surveys conducted across the outer continental shelf and slope of southeast and southern Brazilian coast. Despite no abundance estimates are yet available, the species was frequently sighted across the offshore sampling area and in large groups (mean = 37 individuals; *SE* = 8; Di Tullio et al., 2016). On the other hand, remarkably low levels of genetic diversity for the coastal ecotype likely reflects small population sizes of possibly a few hundred individuals (Fruet, Flores, et al., 2016; Fruet, Zappes, et al., 2016). Mark‐recapture data from long‐term studies of coastal populations along the SWA indicate critical small population sizes (populations not exceeding 90 individuals) and high site fidelity of individuals (e.g., Daura‐Jorge, Ingram, & Simões‐Lopes, 2013; Fruet, Daura‐Jorge, Möller, Genoves, & Secchi, 2015; Giacomo & Ott, 2016; Laporta, Fruet, et al., 2016; Vermeulen & Cammareri, 2009b).

### 4.2 Population structure

We found strong signals of population structure between coastal and offshore bottlenose dolphin ecotypes in the SWA that is consistent with current habitat usage preferences. Ecotypes displayed a great number of private alleles and did not share mtDNA haplotypes, suggesting current and long‐term genetic isolation. This is surprising given the absence of geographical barriers to gene flow in the broad geographical sampling area examined in the present study, which encompasses zones with high potential for gene flow between the ecotypes (i.e., zones of sympatry or where offshore ecotypes are often seen close to the shore). In Bahía San Antonio, for example, Vermeulen and Cammareri (2009a) reported the presence of three morphologically distinct individuals that were observed on a regular basis interacting together with individuals of the small coastal dolphin population of this area. Analyses of both molecular marker types clustered their genetic profile with the offshore ecotype, indicating they are likely migrants from the offshore population. The evidence for genetic isolation between offshore and coastal ecotypes living in sympatry in BSA suggests that complex mechanisms are involved in the genetic structuring of bottlenose dolphins in this region.

Several hypotheses have been proposed to explain processes driving high genetic diversification in species living in environments where there are no geographical barriers to gene flow. For the well‐studied killer whales (*Orcinus orca*), for example, feeding strategies are believed to have played a crucial role in shaping genetic structuring in sympatric and parapatric populations (e.g., Foote et al., 2011). For bottlenose dolphins, despite several hypotheses proposed (e.g*.,* habitat preferences, philopatry to the natal area, vertical transmission of social learning, feeding specialization), there is only one study that explicitly tested for forces driving ecotype differentiation and population structure (Louis, Fontaine, et al., 2014). This study suggested that coastal populations in Northeastern Atlantic (NEA) were founded by pelagic dolphins after the Last Glacial Maximum, perhaps due to emerging opportunities to explore vacant ecological niches. The occupation of these coastal zones would have followed successive events of feeding specialization and natal philopatry, leading to fine‐scale population structuring and a reduction in genetic diversity (e.g., Hoelzel et al., 1998; Louis, Fontaine, et al., 2014; Natoli et al., 2004; Tezanos‐Pinto et al., 2009). This process of diversification is a plausible scenario for bottlenose dolphins in the SWA, which presented similar genetic signals to those found in the North Atlantic (i.e., ecotypes with contrasting levels of genetic diversity and following independent evolutionary trajectories). However, this hypothesis should be explicitly tested exploring the historical demography of ecotypes through coalescent‐based analysis in combination with other ecological and biological data.

In the NEA and wider Caribbean, as well as in the Pacific Ocean, there was no complete lineage sorting despite high genetic differentiation between ecotypes in nuclear and mtDNA markers (Caballero et al., 2012; Louis, Viricel, et al., 2014; Lowther‐Thieleking, Archer, Lang, & Weller, 2015; Segura, Rocha‐Olivares, Flores‐Ramírez, & Rojas‐Bracho, 2006). In the Northwestern Atlantic (NWA), however, current gene flow seems to be trivial between ecotypes, with the coastal haplotypes forming a separate evolutionary lineage (Natoli et al., 2004; Tezanos‐Pinto et al., 2009), similar to what we have found in the present study. In the NWA, the coastal ecotype is highly differentiated in ecology (distribution, feeding ecology, and parasite loads), morphology, and genetics (e.g., Hersh & Duffield, 1990; Mead & Potter, 1990, 1995; Rosel et al., 2009), with restricted distribution to this oceanographic region (Natoli et al., 2004). It was further suggested that the coastal ecotype might, in fact, represent a different species from the offshore ecotype inhabiting this ocean region (see Kingston & Rosel, 2004). For the SWA, little information is available distinguishing both ecotypes. The presence of coastal and offshore ecotypes have been preliminary suggested based on color pattern, feeding ecology, and genetics (Botta, Hohn, Macko, & Secchi, 2012; Costa et al., 2015), and only recently a detailed study based on skull and skeletal morphology of stranded dolphins have demonstrated the presence of two distinct ecotypes living in parapatry (Costa et al., 2016). In addition, the great morphological differentiation between the ecotypes led the later authors to suggest that these groups are distinct subspecies, with the coastal ecotype restricted to inshore waters of the southern coast of the SWA and the offshore ecotype widespread along the continental shelf waters and beyond. The previous study, however, did not examine the potential genetic differentiation between the ecotypes. Our data did not genetically examine the same samples used in Costa et al. (2016), but there is an overlap in the sampling areas. Therefore, if the parapatric distribution suggested is correct, and considering our sampling areas, the results presented here seem to be in agreement with the ecotypes described by Costa et al. (2016). Ongoing analyses testing both nuclear and mitochondrial markers as well as morphology are exploring the congruence between the genetic and morphological data in the attempt to clarify the taxonomic status of bottlenose dolphins in the SWA (A.B.P. Costa, P.F. Fruet, E.R. Secchi, F.G. Daura‐Jorge, P.C. Simões‐Lopes, J.C. Di Tullio, P.E. Rosel unpublished data).

### 4.3 Implications for conservation

Our results from maternal and biparental molecular markers were congruent and showed that coastal and offshore bottlenose dolphin ecotypes in the SWA are genetically distinct and are possibly following discrete evolutionary trajectories. Sighting data from the literature indicates that coastal bottlenose dolphins are restricted to shallow waters of the southern coast of the continent (above 25–27°S), while the offshore ecotype preferentially inhabits deeper waters albeit some incursions to coastal areas can occur occasionally in the north limit of the distribution of the coastal ecotype. Despite opportunity for gene flow in this possible “contact zones” our results suggest negligible interbreeding between ecotypes, even in an area where dolphins of both ecotypes were observed to associate (Vermeulen & Cammareri, 2009a). Based on our findings, which seem to be in agreement with the morphological differentiation described by Costa et al. (2016), we recommend recognizing the offshore bottlenose dolphin ecotype as an additional Evolutionarily Significant Unit (ESU) in the SWA. The recognition of this ESU in the SWA is relevant in the context of planning and prioritizing unit‐specific conservation strategies. Studies should therefore consider the offshore ESU separately for abundance estimates, monitoring, and population assessments. Nevertheless, it is important to point out that the genetic isolation observed in the coastal ESUs (Fruet et al., 2014) increases the risk of inbreeding depression and extinction of the coastal ecotype. This ecotype is restricted to a relatively small area and is currently genetically depauperated, with small population sizes and evidence of increasing threats from several anthropogenic activities (Fruet et al., 2014; see Fruet, Flores, et al., 2016; Fruet, Zappes, et al., 2016 for review) and local population declines (Coscarella, Dans, Degrati, Garaffo, & Crespo, 2012; Vermeulen & Bräger, 2015). Thus, conservation measures to enhance the long‐term viability of this possible endemic ecotype need to be prioritized as previously recommended (see Fruet et al., 2014 for specific recommendations for the conservation of coastal ESUs).

## CONFLICT OF INTEREST

None declared.

## AUTHOR CONTRIBUTION

Pedro F. Fruet contributed substantially to the conception, acquisition of samples, analysis, interpretation and drafting the work; Eduardo R. Secchi contributed substantially to the conception, acquisition of samples of the work, drafting, and revising it critically for important intellectual content; Juliana C. Di Tullio, Fábio G. Daura‐Jorge, Paulo César Simões‐Lopes, Els Vermeulen, Paulo A. C. Flores, Rodrigo Cezar Genoves, and Paula Laporta contributed substantially to the acquisition of samples, drafting, and revising it critically for important intellectual content; Ana P.B. Costa contributed substantially to data analysis, drafting, and revising the work critically for important intellectual content; Luciano B. Beheregaray and Luciana Möller contributed substantially to the conception, data analysis, interpretation, drafting, and revising it critically for important intellectual content.
