# Supplementary material for: Genetic divergence between two phenotypically distinct bottlenose dolphin ecotypes suggests separate evolutionary trajectories
Source: Ecol Evol. 2017 Sep 29;7(21):9131–43. doi: 10.1002/ece3.3335 (PMC5689489; doi:10.1002/ece3.3335)
Supplement: Supplementary file 1 [file ECE3-7-9131-s001.docx]

Figure S1. LnPD (above) and Delta K (below) against K for 20 replicates of STRUCTURE HARVESTER output.
