# Supplementary material for: Genetic divergence between two phenotypically distinct bottlenose dolphin ecotypes suggests separate evolutionary trajectories
Source: Ecol Evol. 2017 Sep 29;7(21):9131–43. doi: 10.1002/ece3.3335 (PMC5689489; doi:10.1002/ece3.3335)
Supplement: Supplementary file 2 [file ECE3-7-9131-s002.docx]

|  |  |  |  |  |  |  |  |  |  |
| --- | --- | --- | --- | --- | --- | --- | --- | --- | --- |
|  |  |  |  |  |  |  |  |  |  |
|  | OFFSHORE | |  | COASTAL | |  | OVERALL | |  |
|  |  |  |  |  |  |  |  |  |  |
| LOCUS | FIS | ONE SIDE P-VALUE |  | FIS | ONE SIDE P-VALUE |  | FIS | ONE SIDE P-VALUE |  |
| *TUR142* | 0.066 | 0.257 |  | 0.956 | 0 |  | 0.419 | 0 |  |
| *TUR91* | 0.248 | 0.003 |  | --- | --- |  | 0.243 | 0.003 |  |
| *TUR141* | 0.312 | 0.039 |  | -0.025 | 0.89 |  | 0.117 | 0.122 |  |
| *TURF10* | 0.088 | 0.156 |  | 0.178 | 0.096 |  | 0.104 | 0.056 |  |
| *TURE12* | 0.035 | 0.397 |  | 0.084 | 0.116 |  | 0.069 | 0.104 |  |
| *TUR105* | -0.046 | 0.356 |  | -0.014 | 0.939 |  | -0.045 | 0.346 |  |
| *TUR80* | 0.069 | 0.281 |  | 0.489 | 0 |  | 0.164 | 0.021 |  |
| *TUR87* | -0.11 | 0.421 |  | -0.002 | 0.995 |  | -0.098 | 0.42 |  |
| *MK6* | 0.043 | 0.214 |  | 0.435 | 0.002 |  | 0.130 | 0.002 |  |
| *MK8* | 0.190 | 0.006 |  | 0.106 | 0.059 |  | 0.134 | 0.004 |  |
| *KW2* | 0.137 | 0.017 |  | 0.265 | 0 |  | 0.225 | 0 |  |
| *KW12* | 0.083 | 0.175 |  | 0.248 | 0.008 |  | 0.176 | 0.003 |  |
| *EV37* | 0.157 | 0.001 |  | 0.117 | 0.093 |  | 0.134 | 0.003 |  |
| *TEXVET* | 0.298 | 0 |  | -0.017 | 0.94 |  | 0.248 | 0 |  |
| *TTR63* | 0.114 | 0.032 |  | 0.183 | 0.023 |  | 0.156 | 0.005 |  |
| *TTR04* | 0.01 | 0.513 |  | 0.109 | 0.022 |  | 0.082 | 0.032 |  |
| Multi-locus | 0.104 | 0 |  | 0.194 | 0 |  | 0.149 | 0 |  |
|  |  |  |  |  |  |  |  |  |  |
|  |  |  |  |  |  |  |  |  |  |

**Table S1. Summary of 16 loci typed for offshore and coastal bottlenose dolphins and their respective estimates of FIS.**
